# Identifying unilateral disease in Chinese patients with primary aldosteronism by using a modified prediction score

**DOI:** 10.1097/HJH.0000000000001488

**Published:** 2017-07-13

**Authors:** Ying Zhang, Wenquan Niu, Fangfang Zheng, Hua Zhang, Wenlong Zhou, Zhoujun Shen, Jianzhong Xu, Xiaofeng Tang, Jin Zhang, Ping-Jin Gao, Ji-Guang Wang, Limin Zhu

**Affiliations:** a Department of Hypertension; b Shanghai Institute of Hypertension, Shanghai Key Laboratory of Hypertension, Ruijin Hospital, Shanghai Jiaotong University School of Medicine, Shanghai; c Department of Cardiology, Tongji Hospital, Tongji University; d Department of Radiology; e Department of Urology of Luwan Branch; f Department of Urology, Ruijin Hospital, Shanghai Jiaotong University School of Medicine, Shanghai, China

**Keywords:** adrenal venous sampling, prediction score, primary aldosteronism, unilateral

## Abstract

**Objective:**

The current study aimed to evaluate the role of Küpers’ score in predicting unilateral aldosteronism, and develop a modified score in Chinese patients with primary aldosteronism.

**Methods:**

The current retrospective study included 406 patients with primary aldosteronism who underwent successful adrenal venous sampling (AVS) and were divided into the unilateral (*n* = 211) and bilateral (*n* = 195) groups according to the AVS results. Normokalemia was noted in both the unilateral (*n* = 64) and bilateral groups (*n* = 84) when plasma and urinary aldosterone were measured.

**Results:**

We evaluated Küpers’ prediction score, which had the best cutoff value at four points [area under the curve, 0.601 (95% confidence interval 0.551–0.650); specificity, 53%; sensitivity, 62%]. Then, we modified this score by using urinary aldosterone level quartiles, history of hypokalemia, and typical adenoma more than 10 mm on computed tomography (CT) [area under the curve, 0.745 (95% confidence interval 0.667–0.813)]; sensitivity, 45.3%; specificity, 90.5%). The best cutoff value to discriminate unilateral from bilateral disease was a score of 5. This modified prediction score only applied to patients who were normokalemic when urinary aldosterone was measured. A specificity of 100% was achieved at a score of 6 for patients aged 40 years or less, and 5 when the adrenal lesion was on the right side on CT imaging.

**Conclusion:**

Küpers’ prediction score is not suitable for our patients. Urinary aldosterone levels combined with a history of hypokalemia are useful to discriminate unilateral from bilateral aldosteronism in patients with typical adenoma on the right adrenal gland on CT or in patients 40 years old or less.

## INTRODUCTION

Primary aldosteronism is becoming one of the most common causes of secondary hypertension [1]. Patients with primary aldosteronism have a higher prevalence of morbidity from cardiovascular and metabolic diseases than those with essential hypertension. Therefore, it is important to identify patients with primary aldosteronism and provide appropriate treatment according to the primary aldosteronism subtype.

The main subtype of primary aldosteronism is bilateral adrenal hyperplasia (BAH), for which mineralocorticoid antagonist treatment is the first choice. Aldosterone-producing adenoma (APA) accounts for nearly 30% of patients with primary aldosteronism and has the most favorable outcome after adrenalectomy; nearly 40% (20–72%) of patients have hypertension cured [2] without antihypertensive medication after such surgeries.

Distinguishing between unilateral hyperaldosteronism and bilateral adrenal disease before adrenalectomy is an important but challenging task. Adrenal venous sampling (AVS) is considered the ‘gold standard’ to confirm lateralization [3,4], with 95% sensitivity and 100% specificity in discriminating between the two main subtypes, compared with traditional adrenal computed tomography (CT). The updated 2016 Endocrine Society primary aldosteronism diagnosis and treatment guidelines recommend that when surgical treatment is feasible and desired by the patient, an experienced radiologist should use AVS to distinguish between unilateral and bilateral adrenal disease [5]. Given the nearly 10% prevalence of primary aldosteronism in the hypertensive population, the number of candidates for AVS should be relatively high. However, AVS is invasive, technically challenging, and not available at all medical centers. Therefore, an easier method for predicting unilateral hyperaldosteronism would decrease the number of candidates for AVS.

In 2012, Küpers *et al.*[6] proposed a clinical prediction score to identify patients with unilateral hyperaldosteronism and bypass AVS before unilateral adrenalectomy; patients with typical Conn's adenoma and a serum potassium concentration less than 3.5 mmol/l or estimated glomerular filtration rate (eGFR) more than 100 ml/min per 1.73 m^2^ do not require AVS. This prediction resulted in a sensitivity of 53% and specificity of 100%. However, it was not validated with other independent patient cohorts. Riester *et al.*[7] found that Küpers’ prediction score only applied to patients less than 40 years old, whereas correct identification unilateral aldosteronism occurred with a sensitivity of 38.8% and specificity of 88.5% in another study with 75 patients, leading the authors to suggest that this prediction score may be useful only for preselecting patients who require AVS instead of replacing AVS [8]. In the current study, we aimed to evaluate Küpers’ score in a much larger cohort of Chinese patients with primary aldosteronism and develop a modified prediction score.

## MATERIALS AND METHODS

### Study design and population

The current retrospective study was performed with a series of patients who were diagnosed with primary aldosteronism between 2005 and 2015 in the Department of Hypertension, Ruijin Hospital, Shanghai, China. The ethics committee of Ruijin Hospital approved the study protocol. All patients provided written informed consent.

The workup for primary aldosteronism complied with the 2008 Endocrine Society clinical guideline for primary aldosteronism [1] and was reported in our previous study [9]. Before and during the workup, patients were advised to follow a diet with regular salt intake and withdraw mineralocorticoid receptor antagonists for at least 6 weeks; nonpotassium sparing diuretics for 4 weeks; and β-blockers, angiotensin-converting enzyme inhibitors, and angiotensin II type 1 receptor blockers for 2 weeks. Nondihydropyridine calcium blockers and/or α_1_-blockers were prescribed for blood pressure (BP) control, as necessary. All patients were hospitalized overnight and blood samples were collected the next morning at approximately 8:00 a.m. Plasma and urinary aldosterone were measured simultaneously with serum electrolytes. All measurements were performed in a College of American Pathologists (no. 7217913) accredited laboratory. Plasma aldosterone concentration (PAC) and plasma renin activity (PRA) were measured by using radioimmunoassay following manufacturer's instructions (Beckman Coulter Corp., Brea, California, USA). The intra-assay and interassay coefficients of variation were 9.3 and 9.5% for aldosterone and 10.1 and 10.2% for renin activity, respectively. The normal range for aldosterone is 2.94–31.33 ng/dl, whereas the normal range for PRA is 0.1–17.4 ng/ml per h.

Patients with baseline serum potassium less than 3.5 mmol/l were given potassium chloride supplementation to ensure that they were normokalemic before confirmation test and AVS. The diagnosis of primary aldosteronism was based on a plasma aldosterone/renin ratio (ARR) activity of more than 24 ng dl/ng ml h by using two independent samples and confirmed with either a fludrocortisone suppression test (FST, before 2007) or a saline infusion test (SIT, from 2007). The protocol for FST was the same as that used by the Brisbane group [10]. Patients received 0.1 mg oral fludrocortisone every 6 h for 4 days, together with sufficient dietary salt and slow-release potassium chloride supplement to insure normokalemia during the testing. On the 4th day, standing PAC and PRA were measured at 1000 h and plasma cortisol concentration was measured at 0700 and 1000 h. Primary aldosteronism was confirmed by using a standing PAC of more than 6 ng/dl, combined with a PRA of less than 1 ng/ml per h, and a plasma cortisol concentration at 1000 h lower than that of the value obtained at 0700 h. From 2007, we switched FST to SIT as the fludrocortisone was no longer available for medical use in China. Patients were infused with 500 ml of 0.9% saline each hour for 4 h from 0800 to 1200 h. during SIT, and primary aldosteronism was confirmed by postinfusion PAC levels more than 6 ng/dl.

### Adrenal venous sampling and computed tomography procedures

All patients first underwent adrenal CT (1.25–3.75 mm/slice) for subtyping. The case was considered typical unilateral APA if a unilateral radiolucent nodule (<10 Hounsfield units on noncontrast) was detected with a diameter more than 10 mm and with normal remaining ipsilateral and contralateral glands. Patients underwent AVS if they were candidates for adrenalectomy according to the clinical manifestations of primary aldosteronism without contraindication for surgery, and if they were willing to receive the operation. AVS was conducted without cosyntropin stimulation and with subsequent catheterization. A selectivity index (defined as the ratio of adrenal venous plasma cortisol concentration to peripheral plasma cortisol concentration) at least 3 was considered to indicate correct catheterization. A lateralization index (defined as the ratio of cortisol-corrected aldosterone from the dominant side to the nondominant side) at least 2 was considered to indicate lateralization.

### Follow-up after adrenalectomy

Adrenalectomy was carried out laparoscopically. Patients who underwent surgery were followed through clinic visits. The data from the last visit were used for analysis. Hypertension was considered cured if SBP/DBP was less than 140/90 mmHg without medication and improved if BP was less than 140/90 mmHg with a reduced number of antihypertensive drugs. Treatment was considered failed if BP was at least 140/90 mmHg with an increase or no change in the number of preoperative antihypertensive drugs.

### Statistical analysis

Statistical analysis was performed by using SPSS Statistics 19.0 (IBM Corp., Armonk, New York, USA). Receiver operating characteristic curve analysis was performed by using MedCalc (version 11.4; MedCalc Software bvba, Ostend, Belgium).

Differences were compared between bilateral AVS and unilateral AVS and considered statistically significant if the *P* value was less than 0.05. Data are expressed as mean ± SD or median (with 25–75th percentile), depending on the distribution. Skewed variables were transformed either logarithmically, or with square transformation. Continuous variables were analyzed by using the Student's *t* test. Categorical variables were compared by using the chi-squared test. Factors that were significantly different between the unilateral and bilateral AVS groups in the univariate analyses (*P* < 0.05) were incorporated into a logistic regression model with a forward-stepwise procedure.

Secondary analyses were conducted with patients with normokalemia when plasma and urinary aldosterone samples were measured as hypokalemia could inhibit the secretion of aldosterone and affect the regression analysis. They were either normokalemic at baseline or after correction with potassium supplements. Factors that were significantly different between the normokalemic unilateral and bilateral AVS groups in the univariate analyses (*P* < 0.05) were incorporated into a logistic regression model with a forward-stepwise procedure.

## RESULTS

Of the 437 patients who were diagnosed with primary aldosteronism and underwent AVS, 211 patients with unilateral hyperaldosteronism and 195 patients with bilateral hyperaldosteronism were included in our analysis (success rate of 93%). When compared with the bilateral-AVS group, greater proportions of the unilateral-AVS group had a history of hypokalemia before hospitalization and potassium chloride supplementation, and the unilateral-AVS group had higher levels of urinary potassium excretion (Table 1). In addition, the unilateral-AVS group had higher plasma aldosterone levels, in both the standing and supine positions, higher urinary aldosterone levels, and higher serum sodium levels. The CT imaging analysis showed that the unilateral-AVS group had a higher proportion of unilateral adenoma more than 10 mm.

### Evaluation of Küpers’ score

The best cutoff value for the Küpers’ prediction score was a score of 4, with an area under the curve (AUC) of 0.601 [95% confidence interval (CI) 0.551–0.650], specificity of 53%, and sensitivity of 62%. With a score of 5 as the cutoff value, which is the optimal Küpers’ score, the specificity reached 82% (95% CI 76–87%), but the sensitivity decreased to 32% (95% CI 26–39%). The positive likelihood ratio was 1.8 (95% CI 1.4–2.2), and the negative likelihood ratio was 0.8 (95% CI 0.6–1.1). eGFR was not significantly different between the two groups.

A history of hypokalemia (*P* = 0.004), serum sodium levels (*P* = 0.007), and supine plasma aldosterone (*P* = 0.011) were entered into the binary logistic regression model (Hosmer–Lemeshow χ^2^ 2.597, *P* = 0.957), the *C*-statistic of which was 0.669 (95% CI 0.613–0.726), with a sensitivity of 65% and specificity of 63%.

In the two groups, 70 and 57% of patients were hypokalemic during the measurement of plasma and urinary aldosterone, respectively, resulting in 64 normokalemic patients in the unilateral-AVS group and 84 normokalemic patients in the bilateral-AVS group were included in the secondary analyses. Among the 148 patients who were normokalemic during the hormonal assessment, 97 (65.5%) patients had a history of hypokalemia, and 51 (34.5%) patients were consistently normokalemic. Greater proportions of the unilateral-AVS group still had a history of hypokalemia and potassium supplementation (Table 2). They also had higher supine plasma aldosterone and urinary aldosterone levels. eGFR was not significantly different between the two groups. A history of hypokalemia (*P* = 0.006) and urinary aldosterone levels (*P* = 0.004) were entered into the logistic regression model (Hosmer–Lemeshow χ^2^ 10.3, *P* = 0.242), and the *C*-statistic was 0.741 (95% CI 0.660–0.822).

### Modified Küpers’ prediction score

**FIGURE 1:**
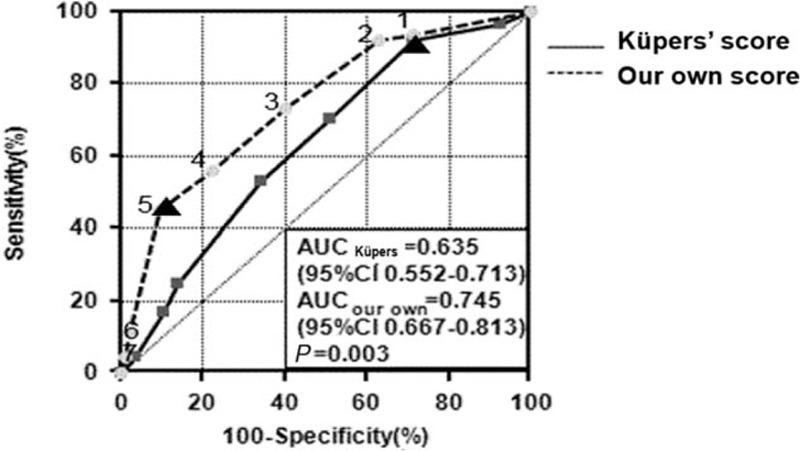
Receiver-operating characteristics curve of our modified prediction score (dashed line) and Küpers’ score (solid line) for unilateral or bilateral hyperaldosteronism in patients whose serum potassium level was normal before the measurement of urinary aldosterone. The best cutoff is identified using triangles. AUC, area under the curve; CI, confidence interval.

**FIGURE 2:**
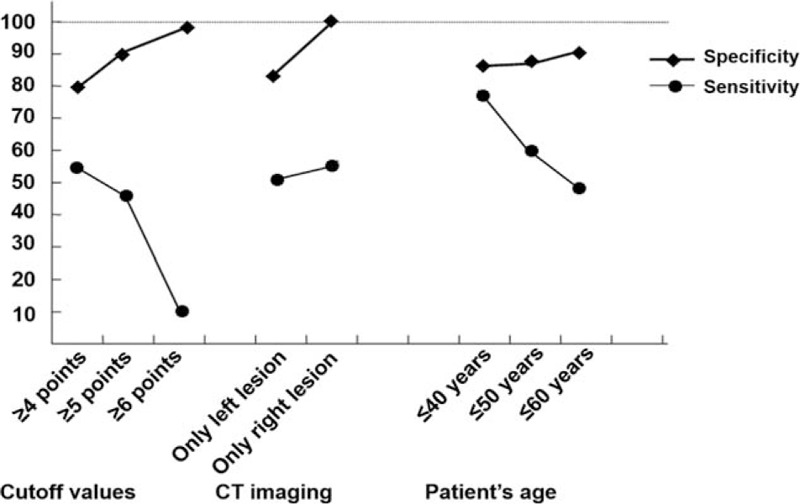
Specificity and sensitivity of our clinical prediction score for unilateral or bilateral hyperaldosteronism based on different cutoff values, computed tomography imaging, and age groups.

The quartiles of urinary aldosterone levels, history of hypokalemia, and typical Conn's adenoma on CT (Table 3) were used to calculate the modified Küpers’ prediction score. We decreased the power of typical adenoma on CT from a score of 3 to 2, given the low concordance between CT imaging and AVS in our cohort (Table S1, Supplemental Digital Content 1, which shows the concordance of CT imaging and AVS results), resulting in a maximum score of 7. The AUC of our modified prediction score was 0.745 (95% CI 0.667–0.813), which is larger than that calculated by Küpers’ rule (0.635, 95% CI 0.552–0.713; *P* = 0.003; Fig. 1). The best cutoff value to discriminate between unilateral and bilateral disease was a score of 5, with a sensitivity of 45.3% (95% CI 32.8–58.3%) and specificity of 90.5% (95% CI 82.1–95.8%). The positive likelihood ratio was 4.8 (95% CI 3.6–6.3), and the negative likelihood ratio was 0.6 (95% CI 0.3–1.2). If we increased the threshold to a score of 6, the specificity reached 97.6%, but the sensitivity decreased to 10.9% (Fig. 2). In the unilateral-AVS group, 29 patients (45%) had a score at least 5; in the bilateral-AVS group, 76 patients (90%) had a score less than 5 (Table S2, Supplemental Digital Content 2, which shows the distribution of score in detail). In total, our modified prediction score could identify that an AVS examination was not necessary for 71% (*n* = 105) of the patients.

We further evaluated the ability of our modified score to identify the side of the unilateral adrenal lesion. We defined a unilateral lesion as being a clear unilateral adrenal adenoma or nodule and a normal contralateral adrenal gland on CT imaging. In patients with a left lesion on CT imaging (*n* = 83), the AUC was 0.697, with a sensitivity of 48.3% and specificity of 85.2%. For patients with a right lesion on CT imaging (*n* = 25), the AUC was 0.873 (95% CI 0.680–0.971), and the best cutoff was a score of 5, with a specificity of 100% (95% CI 71.5–100%) and sensitivity of 56.8% (95% CI 28.9–82.3%) (Fig. 2).

We then evaluated our score in patients younger than 40 years old (*n* = 30). The AUC was 0.810 (95% CI 0.626–0.929) and the best cutoff was a score of 5, the sensitivity was 76.5% (95% CI 50.1–93.2%), and the specificity was 85% (95% CI 54.6–98.1%) (Fig. 2). Adding one point increased the specificity to 100% (95% CI 75.3–100%), but the sensitivity decreased to 23.5% (95% CI 6.8–49.9%). Among 15 patients who had scores higher than 5, 4 had scores higher than 6. Although our score identified only 4 patients with lateral adenoma, it excluded 13 patients with bilateral lesion in this specific group and further helped them avoid receiving the invasive AVS (Table S3, Supplemental Digital Content 3).

Finally, we evaluated our modified score in the initial large cohort of 406 patients. With the best cutoff value of 5 points, the AUC was 0.666 (95% CI 0.612–0.719), and the specificity and sensitivity were 73.9 and 49.0%, respectively.

### Adrenalectomy and follow-up

In the normokalemic subgroup (*n* = 64), 58 patients with lateralized AVS underwent an adrenalectomy and 48 (83%) of them were followed up. Hypertension was cured in 14 patients and BP was controlled with fewer antihypertensive medications in 34 patients. Serum potassium level (4.3 ± 0.3 mmol/l), PRA (2.2 ± 1.5 ng/ml per h), PAC (9.5 ± 3.8 ng/dl), and urinary aldosterone concentration (3.7 ± 2.1 μg/24 h) normalized after surgery for all patients. Twenty-one patients with unilateral adrenal lesions on CT imaging presented with prediction scores of at least 5, indicating that they might not have required AVS before the adrenalectomy.

## DISCUSSION

Based on our evaluation of Küpers’ prediction score in the present sample of 406 patients with primary aldosteronism who underwent AVS, the best cutoff value was a score of 4, with an AUC of 0.601 (95% CI 0.551–0.650), a specificity of 53%, and sensitivity of 62%. This finding suggests that Küpers’ prediction score is not suitable for Chinese patients with primary aldosteronism. We modified the score by using the history of hypokalemia, typical adrenal adenoma on CT imaging, and quartiles of urinary aldosterone levels instead of eGFR, resulting in an AUC of 0.745 (95% CI 0.667–0.813), a sensitivity of 45.3%, and specificity of 90.5%. The best cutoff value to discriminate between unilateral and bilateral disease was 5 points, only for normokalemic patients with simultaneous urinary aldosterone measurement. The specificity was 100% for a score of 6 in patients 40 years old or less and for a score of 5 when the adrenal lesion was on the right side on CT imaging.

There are several possible reasons why the Küpers’ score is not suitable for predicting lateralization in our patient cohort. First, the score includes eGFR, which was quite different between patients with lateralized AVS and nonlateralized AVS in the study by Küpers *et al.*[6] and in the study by Riester *et al.*[7]. However, we did not find this difference in patients with normokalemia or hypokalemia. Second, in the study by Küpers *et al.*, the concordance rate between the results of CT imaging and AVS was nearly 68%, compared with only approximately 40% in the present study, which is as low as that reported by Lim *et al.*[11]. This may be due to thicker slice-cutting used in the early period that decreased the ability to identify small lesions on CT imaging [12]. Finally, an enlarged left adrenal gland on CT imaging was more probably regarded as an adenoma [13]. In the current study, 325 patients who underwent AVS had a left lesion on CT imaging, but only 49.9% (*n* = 202) of the patients had AVS-confirmed left hyperaldosteronism.

Hypokalemia caused by aldosteronism is a typical clinical presentation in patients with primary aldosteronism, with a frequency of 9–37% [14], and the prevalence of hypokalemia differs between APA (48–59%) and BAH (27.5–17%) [15,16]. However, in Chinese patients with primary aldosteronism, the prevalence of hypokalemia is much higher (52.5%) [17], and it reached 79.3% in the patients who underwent AVS in the current cohort. The effect of hypokalemia on the inhibition of aldosterone secretion is more potent than the counter-effect of volume depletion on promoting aldosterone secretion [18]. As nearly 64% patients had hypokalemia during the measurement of plasma and urinary aldosterone levels in the current study, we selected patients who were normokalemic during the measurement, helping to reveal the actual aldosterone level and resulting in the ability to include the urinary aldosterone level in the regression model. The Hosmer–Lemeshow test showed this model was better fitted with a greater discriminative ability (*C*-statistic, 0.741) than the model presented by Küpers *et al.*

There is a close association between 24-h urinary aldosterone levels and left ventricular diastolic dysfunction [19], and 24-h urinary aldosterone levels may predict left ventricular hypertrophy [20] in patients with primary aldosteronism. In a study by Kobayashi *et al.*[21], 24-h urinary aldosterone level was suggested to effectively discriminate unilateral adrenal hyperplasia from bilateral hyperplasia when a cutoff value of 14.5 μg/24 h was used, with a sensitivity of 75.9% and specificity of 88.9%. Therefore, urinary aldosterone level is an important and reasonable component in our modified prediction score.

Our modified score would help some patients to avoid the invasive AVS procedure. Patients with a typical adenoma (>10 mm) on the right adrenal gland on CT imaging are prone to unilateral hyperaldosteronism with a 24-h urinary aldosterone level at least 23 μg/24 h or if they have a combination of a history of hypokalemia and urinary aldosterone level at least 13 μg/24 h. Patients younger than 40 years could also undergo surgery without AVS if they have a typical adenoma on CT, experienced hypokalemia, and have a urinary aldosterone level at least 19 μg/24 h.

Our study has some limitations. First, our modified score performed well only under normokalemic conditions. Despite the initial large number of patients, only 148 normokalemic patients could be analyzed. We used the initial value for serum potassium level, which was measured simultaneously with ARR and urinary aldosterone, to define the patient's potassium status. This resulted in high proportions of hypokalemia patients in both groups (70 and 57%, respectively) though all hypokalemic patients reached eukalemic state before further confirmation test and the AVS procedure. We found that the presence of hypokalemia diminished the power of aldosterone in identifying unilateral aldosteronism. Therefore, it is crucial to correct hypokalemia before the aldosterone measurement during the workup of primary aldosteronism. Second, we used sequential AVS without cosyntropin stimulation in our center, and we employed a lateralization index at least as an indicator of adrenalectomy. However, the procedure was conducted by a highly experienced radiologist with an experience of more than 1000 AVS and a total success rate of nearly 99%. The lateralization index is relatively low, which may select a subset of patients with a lower possibility of being cured from primary aldosteronism by adrenalectomy; however, it complied with the 2014 AVS experts’ consensus [4]. Our primary aldosteronism cure rate was 100% and hypertension cure rate was 29% (14/48), which corresponded with the rates reported by Muth *et al.*[2]. Finally, urinary aldosterone exhibited a marked intraindividual variability [22], and a 24-h urine sample collection is not always convenient, potentially limiting its use in the clinical situation.

In conclusion, Küpers’ prediction score was not suitable for the present Chinese patients with primary aldosteronism. Our modified prediction score, which includes urinary aldosterone stratification, history of hypokalemia, and CT imaging, is useful to discriminate unilateral from bilateral hyperaldosteronism in normokalemic patients by using a cutoff score of 5. AVS is not necessary in patients with a typical right adrenal lesion on CT imaging and a score at least 5, or in patients younger than 40 years with a score at least 6. This modified score needs further evaluation in other larger cohorts.

## ACKNOWLEDGEMENTS

The study was supported by grant 20134109 from the Shanghai Municipal Health Bureau, Shanghai, China and grant 81500324 from National Natural Science foundation of China.

### Conflicts of Interest

There are no conflicts of interest.

## Supplementary Material

Supplemental Digital Content

## Figures and Tables

**TABLE 1:** Baseline clinical characteristics of patients with unilateral or bilateral adrenal venous sampling results

Variable	Unilateral AVS, *n* = 211	Bilateral AVS, *n* = 195	*P* value
Age (years)	48.9 ± 11.0	49.1 ± 11.0	0.845
Sex (male)	128 (60.7)	114 (58.5)	0.686
Hypertension duration (years)	8 (3–13)	10 (3–15)	0.195
Number of antihypertensive drugs	3 (2–3)	3 (2–3)	0.725
SBP (mmHg)	146 ± 17	148 ± 21	0.334
DBP (mmHg)	88 ± 13	88 ± 13	0.683
History of hypokalemia	185 (87.7)	137 (70.3)	<0.001
Lowest potassium level (mmol/l)	2.8 (2.4–3.1)	2.9 (2.4–3.2)	0.218
Serum potassium level (mmol/l)	3.2 ± 0.5	3.4 ± 0.5	<0.001
Serum sodium level (mmol/l)	141.1 ± 2.8	140.1 ± 2.7	0.001
KCL supplementation	152 (72.4)	113 (57.9)	0.003
eGFR (ml/min per 1.73 m^2^)	98.7 (83.6–117.8)	96.7 (83.9–111.4)	0.547
24-h urinary Na^+^ level (mmol/24 h)	155.7 ± 72.5	150.5 ± 68.0	0.476
24-h urinary K^+^ level (mmol/24 h)	57.3 (41.4–80.7)	45.5 (34.0–69.3)	0.002
Supine PAC (ng/dl)	28.8 (19.1–39.4)	21.1 (13.8–30.5)	<0.001
Standing PAC (ng/dl)	27.0 (19.4–36.2)	24.8 (16.7–33.4)	0.047
Standing PRA (ng/ml per h)	0.36 (0.18–0.98)	0.50 (0.22–1.04)	0.340
ARR (ng dl/ng ml h)	63.6 (26.9–154.9)	47.4 (21.7–122.6)	0.119
24-h urinary aldosterone level (μg/24 h)	19.2 (12.6–26.9)	13.5 (8.9–21.6)	<0.001
Typical adenoma ≥10 mm on CT	68 (32)	42 (22)	0.015

Data are expressed as mean ± SD, median (interquartile range), or *n* (%). ARR, aldosterone-to-renin ratio; AVS, adrenal venous sampling; CT, computed tomography; eGFR, estimated glomerular filtration rate; KCL, potassium chloride; PAC, plasma aldosteronism concentration; PRA, plasma renin activity.

**TABLE 2:** Baseline clinical characteristics of patients with normokalemia with unilateral or bilateral adrenal venous sampling results

Variable	Unilateral AVS, *n* = 64	Bilateral AVS, *n* = 84	*P*
Age (years)	48.6 ± 12.1	50.7 ± 10.2	0.248
Sex (male)	43 (65.2)	51 (58.0)	0.365
Hypertension duration (years)	6 (2–11)	10 (3–17)	0.064
Number of antihypertensive drugs	3 (2–4)	3 (2–4)	0.880
SBP (mmHg)	143 ± 16	147 ± 20	0.166
DPB (mmHg)	87 ± 13	87 ± 13	0.810
History of hypokalemia	56 (84.8)	46 (52.3)	<0.001
Lowest potassium level (mmol/l)	2.7 (2.4–3.1)	2.9 (2.5–3.2)	0.448
Serum potassium level (mmol/l)	3.8 ± 0.2	3.9 ± 0.3	0.004
Serum sodium level (mmol/l)	140.7 ± 3.2	139.8 ± 2.9	0.060
KCL supplementation	37 (56.9)	34 (38.6)	0.029
eGFR (ml/min per 1.73 m^2^)	97.9 ± 26.6	93.8 ± 16.8	0.272
24-h urinary Na^+^ level (mmol/24 h)	170.9 ± 82.2	146.3 ± 64.1	0.052
24-h urinary K^+^ level (mmol/24 h)	66.3 ± 33.0	50.8 ± 24.2	0.003
Supine PAC (ng/dl)	29.4 ± 18.2	20.4 ± 10.6	<0.001
Standing PAC (ng/dl)	27.3 ± 15.7	23.5 ± 11.0	0.081
Standing PRA (ng/ml per h)	0.43 (0.23–1.13)	0.61 (0.29–1.22)	0.314
ARR (ng dl/ng ml h)	45.2 (20.0–135.1)	32.2 (18.0–69.7)	0.175
24-h urinary aldosterone level (μg/24 h)	18.3 (10.5–27.7)	12.1 (8.1–16.8)	<0.001
Typical adenoma ≥10 mm on CT	18 (27)	17 (19)	0.244

Data are expressed as mean ± SD, median (interquartile range), or *n* (%). ARR, aldosterone-to-renin ratio; AVS, adrenal venous sampling; CT, computed tomography; eGFR, estimated glomerular filtration rate; KCL, potassium chloride; PAC, plasma aldosteronism concentration; PRA, plasma renin activity.

**TABLE 3:** Modified prediction score for unilateral or bilateral hyperaldosteronism

Item	Points
Urinary aldosterone level (μg/24 h)	
<13	0
13–19	1
19–23	2
≥23	3
History of hypokalemia	2
Typical adenoma ≥1 cm on computed tomography	2

## Reviewer's Summary Evaluation

### Reviewer 1

This is an interesting clinical study proposing a modified prediction score for disease lateralization in patients with primary aldosteronism. Zhang *et al.* evaluated 406 patients with primary aldosteronism and found that a combination of urinary aldosterone, hypokalemia history, and typical adenoma (1cm) on computed tomography might predict unilateral disease in young (<40 years) patients or patients with right adrenal lesion.

Strengths

1. Large number of participants
2. Clinically meaningful finding when adrenal venous sampling is not available or feasible

Limitations

1. Limited generalization of study findings (only Chinese patients included, prediction only for young patients or patients with right adenoma)
