# Supplementary material for: Identifying unilateral disease in Chinese patients with primary aldosteronism by using a modified prediction score
Source: J Hypertens. 2017 Jul 13;35(12):2486–92. doi: 10.1097/HJH.0000000000001488 (PMC5673302; doi:10.1097/HJH.0000000000001488)
Supplement: Supplemental Digital Content [file jhype-35-2486-s001.doc]

**Table S1.** **Concordance of computed tomography (CT) imaging and adrenal venous sampling (AVS) results in 148 normokalemic patients**

|  |  | **CT imaging** | | |
| --- | --- | --- | --- | --- |
|  |  | **Left lesion** | **Right lesion** | **Bilateral lesion** |
| **AVS Results** | **Unilateral left** | 23 | 2 | 10 |
|  | **Unilateral right** | 5 | 13 | 11 |
|  | **Bilateral** | 54 | 11 | 19 |

AVS, adrenal venous sampling; CT, computed tomography

**Table S2. The distribution of our score to predict the results of adrenal venous sampling in 148 normokalemic patients**

| **AVS results** | **Our clinical prediction score** | | | | | | | |
| --- | --- | --- | --- | --- | --- | --- | --- | --- |
|  | 0 | 1 | 2 | 3 | 4 | 5 | 6 | 7 |
| Unilateral left | 3 | 1 | 5 | 7 | 2 | 13 | 2 | 1 |
| Unilateral right | 1 | 0 | 7 | 4 | 5 | 9 | 2 | 2 |
| Bilateral | 24 | 7 | 19 | 15 | 11 | 6 | 1 | 1 |

AVS, adrenal venous sampling

**Table S3. The distribution of modified score in the prediction of lateral adrenal adenoma in patients < 40 years.**

| **AVS results** | **Our clinical prediction score** | | | | | | | |
| --- | --- | --- | --- | --- | --- | --- | --- | --- |
|  | 0 | 1 | 2 | 3 | 4 | 5 | 6 | 7 |
| Unilateral left | 1 | 0 | 1 | 1 | 1 | 5 | 0 | 1 |
| Unilateral right | 0 | 0 | 0 | 0 | 0 | 4 | 2 | 1 |
| Bilateral | 2 | 1 | 1 | 3 | 4 | 2 | 0 | 0 |

AVS, adrenal venous sampling
